# The transcriptional coregulator *NAB2* is a target gene for the Wilms' tumor gene 1 protein (WT1) in leukemic cells

**DOI:** 10.18632/oncotarget.19896

**Published:** 2017-08-03

**Authors:** Helena Jernmark Nilsson, Giorgia Montano, Tove Ullmark, Andreas Lennartsson, Kristina Drott, Linnea Järvstråt, Björn Nilsson, Karina Vidovic, Urban Gullberg

**Affiliations:** ^1^ Division of Hematology and Transfusion Medicine, Department of Laboratory Medicine, Lund University, Lund, Sweden; ^2^ Department of Biosciences and Nutrition, Karolinska Institutet, Huddinge, Sweden

**Keywords:** WT1, NAB2, target gene, coregulator, leukemia

## Abstract

The Wilms’ tumor gene 1 (*WT1*) is recurrently mutated in acute myeloid leukemia. Mutations and high expression of *WT1* associate with a poor prognosis. In mice, WT1 cooperates with the *RUNX1/RUNX1T1* (*AML1/ETO*) fusion gene in the induction of acute leukemia, further emphasizing a role for WT1 in leukemia development. Molecular mechanisms for WT1 are, however, incompletely understood. Here, we identify the transcriptional coregulator *NAB2* as a target gene of WT1. Analysis of gene expression profiles of leukemic samples revealed a positive correlation between the expression of *WT1* and *NAB2*, as well as a non-zero partial correlation. Overexpression of *WT1* in hematopoietic cells resulted in increased *NAB2* levels, while suppression of *WT1* decreased *NAB2* expression. WT1 bound and transactivated the proximal *NAB2* promoter, as shown by ChIP and reporter experiments, respectively. ChIP experiments also revealed that WT1 can recruit NAB2 to the *IRF8* promoter, thus modulating the transcriptional activity of WT1, as shown by reporter experiments. Our results implicate *NAB2* as a previously unreported target gene of WT1 and that NAB2 acts as a transcriptional cofactor of WT1.

## INTRODUCTION

The Wilms’ tumour gene 1 transcription factor (*WT1*) was first identified as a tumour suppressor gene in Wilms' tumour [1, 2]. However, only a fraction of Wilms' tumours have *WT1* inactivating mutations, and in other cases *WT1* rather shows overexpression [3]. *WT1*-null mice die *in utero* with severe malformations of the urogenital system, heart, lungs, and diaphragm [4], indicating *WT1* as critical for development of several organs [5]. The Denys-Drash syndrome, caused by germ line mutations of *WT1*, shows the importance of *WT1* for human renal and genital development [6].

In the hematopoietic system, *WT1* is normally expressed in a small subset of CD34^+^ progenitor cells [7–9] and murine hematopoietic stem cells lacking *WT1* are incapable of competing with normal stem cells in reconstitution [10]. Inducible deletion of *WT1* in adult mice causes aberrant hematopoiesis, with complete failure of erythrocyte formation due to intrinsic defects of erythroid progenitors [11]. Additionally, *WT1* appears to have an oncogenic role in hematologic malignancies [12]. In most acute myeloid leukemia (AML) and acute lymphoid leukemia (ALL) blast cells, *WT1* is highly expressed and correlated to poor clinical outcome [13–16]. Presence of acquired somatic *WT1* gene mutations is an independent negative prognostic marker, found in 10-15% of cytogenetically normal AML (CN-AML) at diagnosis [17]. Upon forced overexpression, the WT1 protein interferes with differentiation of myeloid cell lines [18–20] and cooperates with the fusion protein RUNX1/RUNX1T1 (AML1/ETO) in a rapid induction of leukemia in transgenic mice, clearly demonstrating a leukemogenic role for *WT1* [21]. Thus, clinical, as well as experimental, data indicate the importance of *WT1* as an oncogene in leukemogenesis. Molecular mechanisms by which WT1 protein exerts its function are, however, incompletely understood.

The EGR (Early Growth Response) transcription factors belong to the same family of Cys_2_His_2_ zinc-finger proteins as WT1, and EGR1 and WT1 share overlapping DNA-binding sites in some promoters [22–23]. The corepressor *NAB2* (NGFI-A Binding Protein 2) is transcriptionally induced by EGR1 [18] and NAB2 acts in a feed-back loop by direct interaction with EGR1, affecting its transcriptional activity [24–26].

Given the similarities in the DNA-binding specificity of WT1 and EGR1, we hypothesized that *NAB2* is a target gene also of WT1. Here, we report that WT1 binds to and trans-activates the *NAB2* promoter and that NAB2 binds to WT1 and modulates its transcriptional activity.

## RESULTS

### Forced expression of *WT1* in hematopoietic cells induces *NAB2* expression

**Figure 1:**
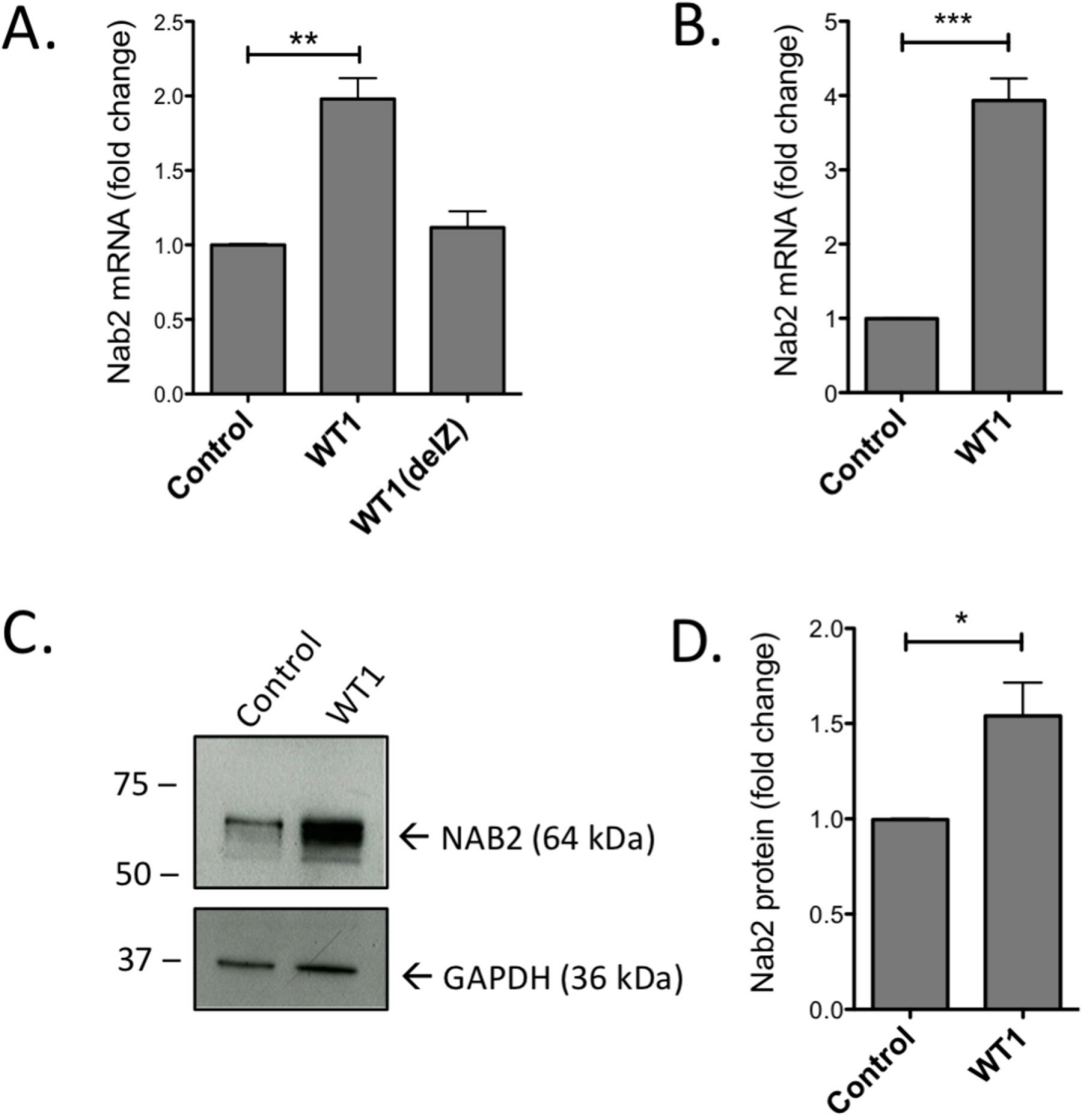
Overexpression of WT1 induces increase of NAB2 mRNA and protein CD34+ progenitor cells or U937 cells were retrovirally transduced with a vector encoding full length WT1+/− isoform (WT1), WT1 lacking zinc fingers (WT1(delZ)), or with empty vector (Control). After transduction and sorting for GFP+ cells, RNA and protein were extracted and analyzed by qPCR and immunoblotting, respectively. Shown are relative levels of *NAB2* mRNA in CD34+ cells **(A)** and in U937 cells **(B)**, immunoblotting of NAB2 protein in U937 cells **(C)**, and protein levels, as determined by densitometry **(D)**. Mean values, bars ±S.E.M., *n* = 3. Stars indicate statistical significance (^*^: p < 0.05; ^**^: p < 0.01; ^***^: p < 0.001).

Normal CD34^+^ progenitor cells, with undetectable or very low expression of endogenous WT1, were retrovirally transduced with *WT1* encoding the WT1+17AA-KTS isoform (WT1+/−), since -KTS isoforms are considered as the most efficient DNA-binders [27]. Forty-eight hours after transduction, GFP+ cells were selected for by sorting, and RNA was analyzed by RT-qPCR. As shown in Figure 1A, *NAB2* expression was induced by WT1, but not by zinc-finger deleted WT1 (WT1(delZ)), demonstrating the dependence on the DNA-binding zinc-fingers of WT1. Similar results with raised *NAB2* mRNA levels (Figure 1B) and NAB2 protein (Figure 1C, D) were obtained upon transduction of the leukemic U937 cell line (lacking endogenous expression of *WT1*) with *WT1*. These results suggest that WT1 binds to and transactivates the *NAB2* gene.

### Suppression of endogenous *WT1* reduces expression of *NAB2*

**Figure 2:**
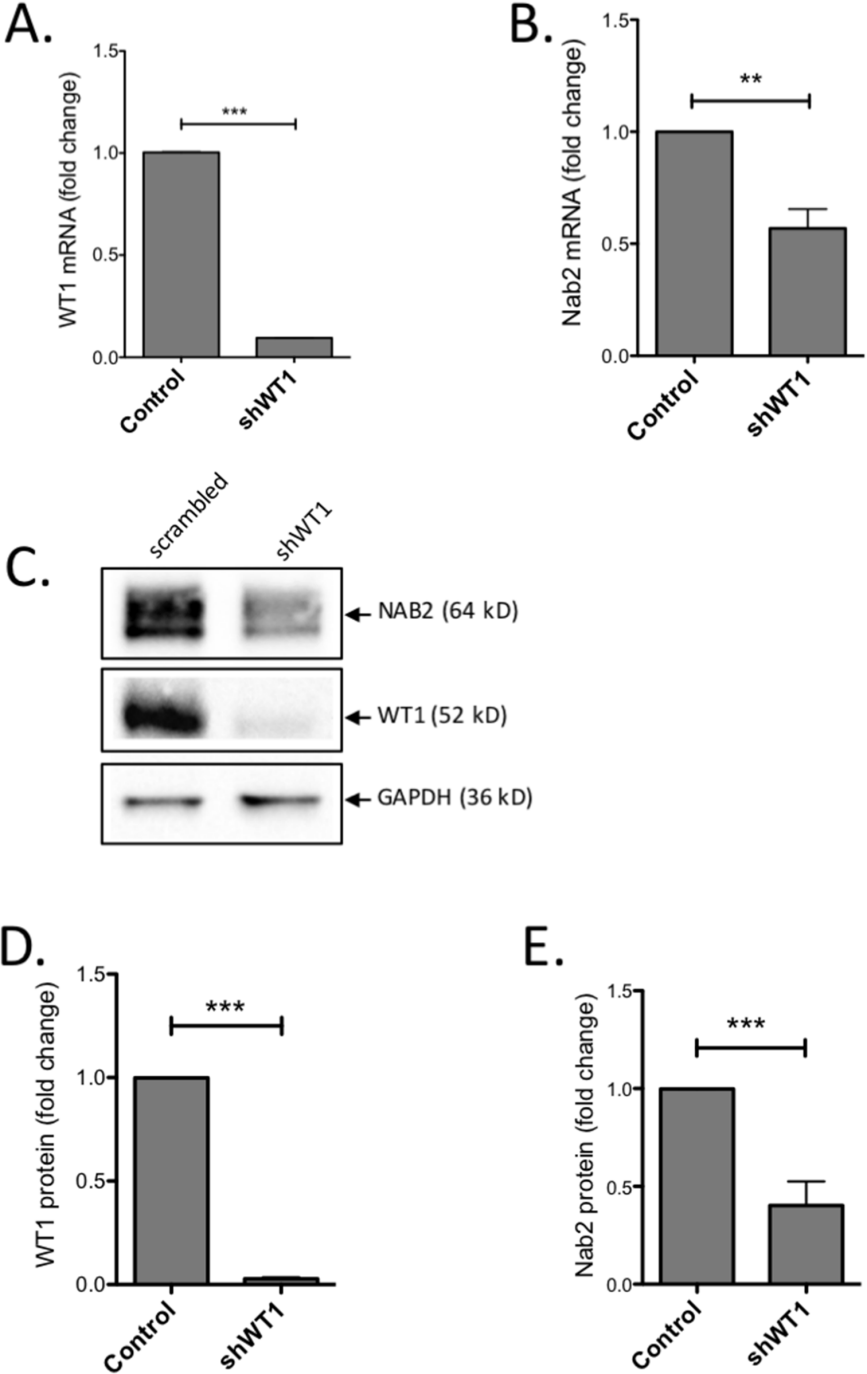
Knock-down of WT1 induces reduction of NAB2 mRNA and protein K562 cells were transduced with a lentiviral vector expressing an shRNA directed against *WT1*, or with scrambled shRNA as control. After transduction and selection with puromycin, RNA and protein were extracted and analyzed by qPCR and immunoblotting, respectively. Shown are relative levels of *WT1* mRNA **(A)** and *NAB2* mRNA **(B)**, immunoblotting of NAB2 and WT1 protein **(C)**, and protein levels, as determined by densitometry **(D, E)**. Mean values, bars ±S.E.M., *n* = 3. Stars indicate statistical significance (^**^: p < 0.01; ^***^: p < 0.001).

To investigate the importance of WT1 for *NAB2* expression further, we employed leukemic K562 cells which have endogenous expression of both *WT1* and *NAB2*. K562 cells were transduced with a lentiviral shRNA vector, previously used to efficiently repress *WT1* expression [28]. After puromycin selection for shRNA-expressing cells, RNA and protein levels were analyzed. As expected, K562 cells expressing *WT1*-shRNA showed a pronounced reduction of *WT1* mRNA and protein (Figure 2A, C, D), as compared to control cells. This reduction of WT1 was paralleled by a decrease of *NAB2* mRNA and protein levels (Figure 2B, C, E). These results corroborate the importance of WT1 for *NAB2* expression in hematopoietic cells.

### The *WT1* and *NAB2* expression correlates in leukemia

We next tested for correlations between *WT1* and *NAB2* expression in sample sets of gene expression profiles of primary AML and CML (chronic myeloid leukemia) samples (Materials and Methods). We observed a positive correlation across all tested data sets between the expression of *WT1* and *NAB2*, (average *r*=0.36), as well as a non-zero partial correlation between *WT1* and *NAB2* (0.046 with Ultranet; rank 5 out of 20,311 genes in the model; [29]). The non-zero value indicates that the correlation between them cannot be explained in full by co-correlation with other observed variables [29]. These data further support that WT1 acts as a transcriptional activator of *NAB2* in leukemic cells.

### WT1 activates the *NAB2* promoter in leukemic cells

**Figure 3:**
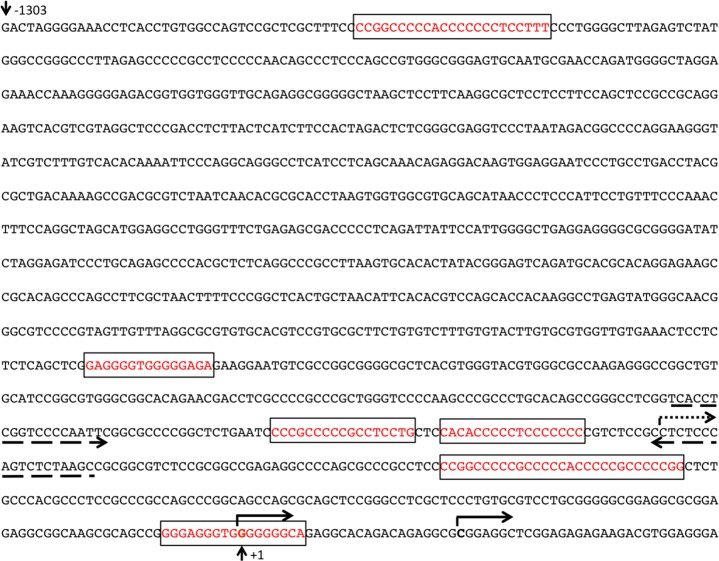
The *NAB2* promoter Solid arrows indicate two alternative transcription start sites (TSSs) in leukemic cells, as determined by CAGE analysis [30]. Dotted arrow indicates the previously identified TSS (NCBI Reference Sequence: NM_005967.3). Dashed arrows indicate the primers used for ChIP experiments. Numbering relates to the upstream TSS in leukemic cells (+1). Boxes indicate putative WT1-binding regions.

**Figure 4:**
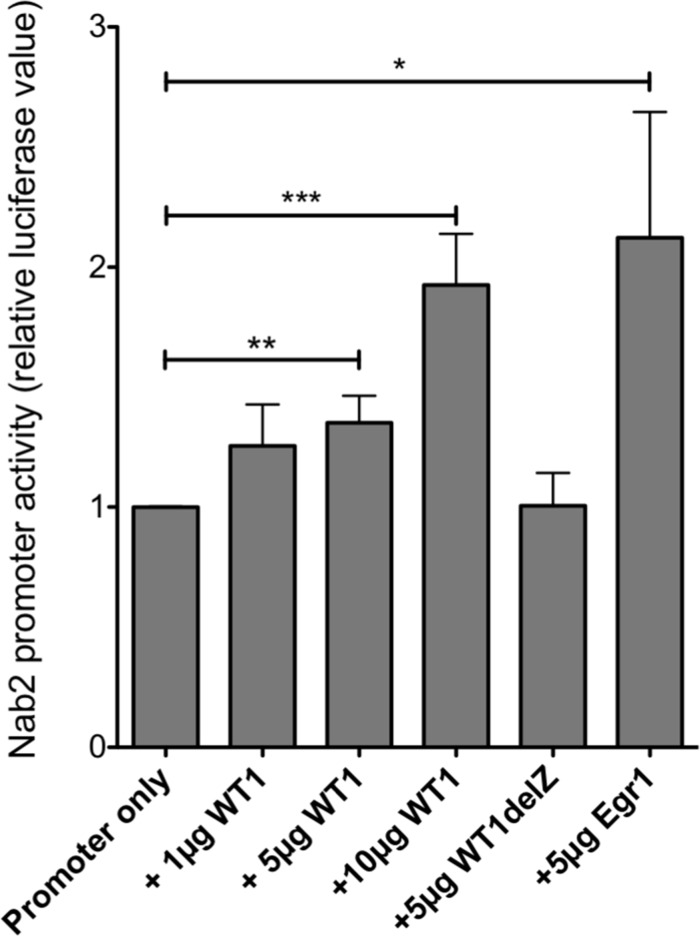
WT1 activates the *NAB2* promoter A reporter plasmid containing 1,300 bp *NAB2* promoter sequence (Figure 3) was cotransfected by electroporation, with different amounts of expression plasmids encoding WT1+/−, WT1(delZ) or EGR1 into U937 cells, as indicated. Shown are normalized luciferase levels, relative to those obtained with promoter reporter only. Mean values, bars ±S.E.M., *n* = 4-7. Stars indicate statistical significance (^*^: p < 0.05; ^**^: p < 0.01; ^***^: p < 0.001).

In carcinoma cells, a GC-rich *NAB2* proximal promoter located within 600 bp upstream of the transcription start site (TSS) has been functionally defined, including several Egr1/Sp1 response elements [24]. Upon inspection of data from CAGE analysis [30], we identified two closely located transcription start sites utilized in leukemic cells, positioned within the previously defined exon 1 (NCBI Reference Sequence: NM_005967.3), confirming the location of the proximal promoter. Upon bioinformatic examination using the Matinspector software (*https://www.genomatix.de*), several potential WT1-binding sites were identified in this area. A luciferase reporter plasmid with a *NAB2* genomic fragment of approximately 1,300 bp upstream of the TSSs, containing six putative WT1 response elements (Figure 3) was transfected into WT1 negative leukemic U937 cells, after which luciferase activity was determined. As shown in Figure 4, cotransfection with increasing amounts of *WT1*+/− resulted in increased promoter activation, while *WT1(delZ)*, lacking the DNA-binding zinc-fingers, was without any effect. Consistent with previous data [24], transfection with *EGR1* also activated the promoter, confirming EGR1 as a positive regulator of *NAB2* (Figure 4). These results indicate that WT1 can bind to the *NAB2* promoter, resulting in enhanced transcription.

### WT1 binds to the *NAB2* promoter *in vivo*

**Figure 5:**
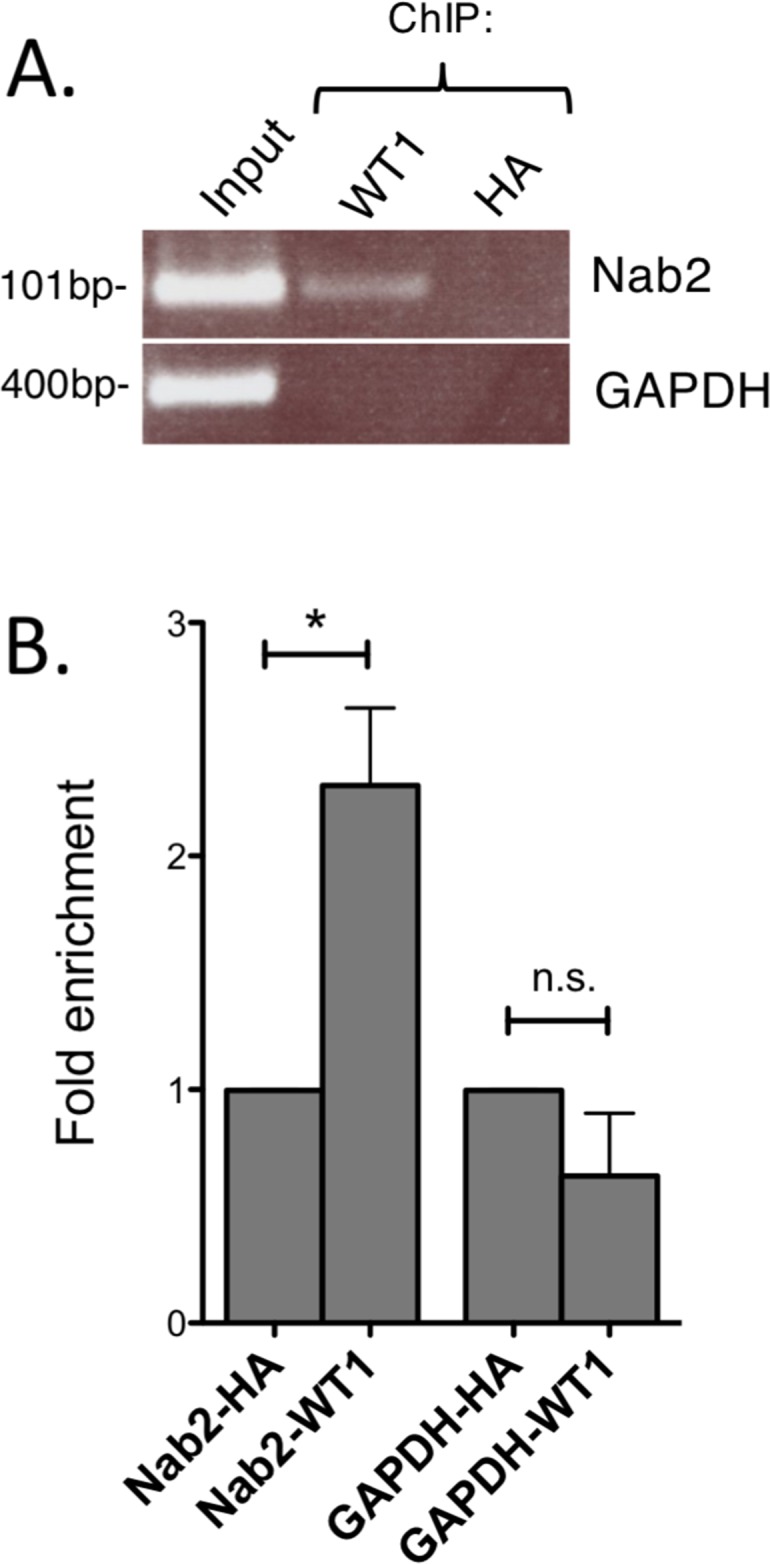
WT1 binds to the *NAB2* promoter Nuclear extracts were obtained from K562 cells and cross-linked chromatin was prepared. **(A)** Chromatin immunoprecipitation (ChIP) using an antibody against WT1 (F6, Santa Cruz) and with anti-HA antibody as negative control. PCR amplification of precipitated DNA was done with primers specific for the *NAB2* promoter or for the *GAPDH* promoter as negative control. **(B)** Fold enrichment as determined by densitometry. Mean values, bars ±S.E.M., *n* = 3. Star indicates statistical significance (^*^: p < 0.05), *n.s*.: not significant.

To find further evidence for WT1 binding to the *NAB2*-promoter, chromatin immunoprecipitation (ChIP) experiments were performed. Chromatin from K562 cells, with endogenous expression of *WT1* and *NAB2*, was subjected to immunoprecipitation with anti-WT1 antibody, after which DNA of the *NAB2* promoter was amplified by PCR with PCR-primers flanking two potential WT1 sites positioned in tandem (Figure 3). Amplification of the *GAPDH* promoter was used as negative control. As shown in Figure 5, precipitation with anti-WT1 resulted in enrichment of the *NAB2* promoter signal, as compared to signal obtained after precipitation with anti-HA, used as negative control. Lack of enrichment of the *GAPDH* promoter signal indicated specificity (Figure 5). We conclude that WT1 binds to the *NAB2* promoter *in vivo*.

### NAB2 binds to WT1

**Figure 6:**
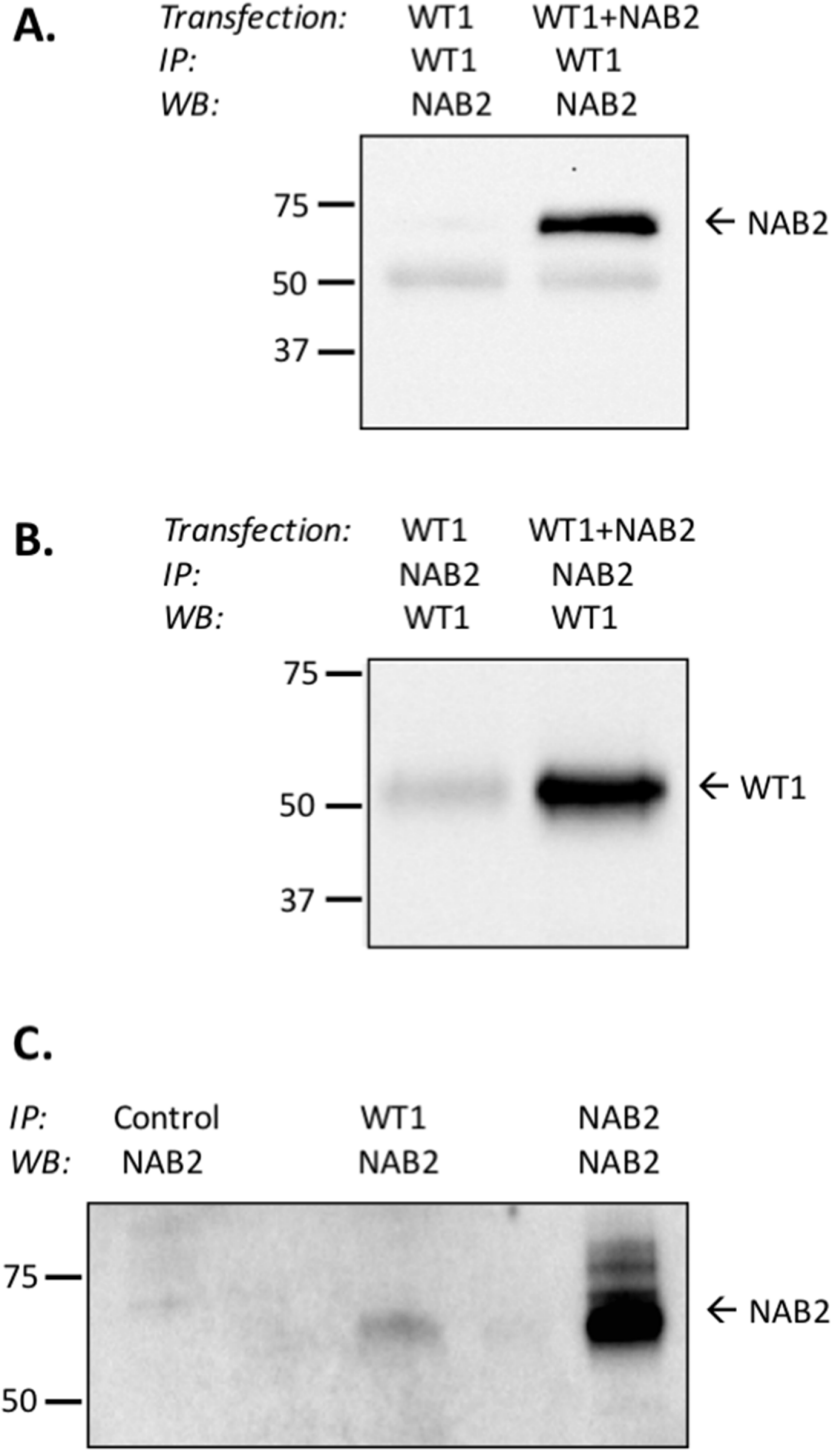
WT1 binds to NAB2 **(A, B)** 293T/17 cells were transfected with *WT1* alone or with *NAB2*, as indicated. After 48 hours nuclear extracts were prepared, from which WT1 (Santa Cruz, C-19) or NAB2 (Abcam, 1C4) was immunoprecipitated (IP), as indicated. Coprecipitated NAB2 (A) or WT1 (B) was detected by immunoblotting (WB), using anti-WT1 (Santa Cruz, sc-192, clone C19), or anti-NAB2 (Abcam, ab135665). **(C)** From nuclear extracts of K562 cells, immunoprecipitation was performed with Sepharose only (negative control), with anti-WT1, or with anti-NAB2 (positive control), followed by immunoblotting (WB) with anti-NAB2. The Clean-blot IP detection kit (HRP) (Thermo Scientific) was utilized to eliminate detection-interference from heavy-chain and light-chain IgG-fragments.

NAB2 and EGR1 proteins bind directly to each other [24, 25]. To investigate if NAB2 also binds to WT1, 293T/17 cells were transfected with *WT1*+/− and *NAB2*, after which coimmunoprecipitation, followed by immunoblotting was performed. As seen in Figure 6A, NAB2 was coprecipitated with WT1, and likewise, WT1 was coprecipitated together with NAB2 (Figure 6B). NAB2 and WT1 interacted with each other also at physiological protein levels, as shown by coprecipitation using K562 cells with endogenous expression of both proteins (Figure 6C). These data indicate that WT1 and NAB2 bind to each other, which is consistent with a functional interaction.

### NAB2 counteracts WT1-mediated transcriptional repression

**Figure 7:**
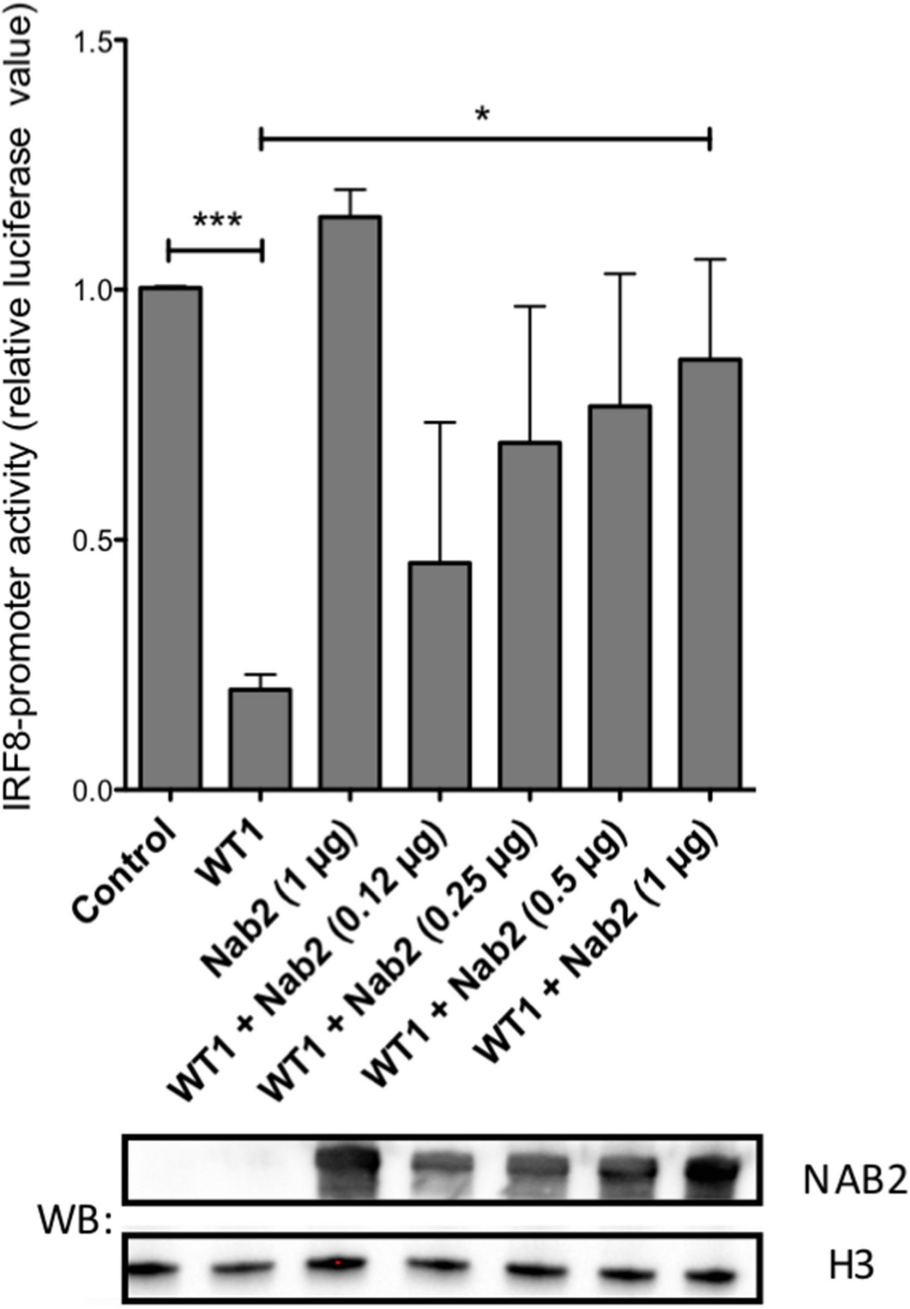
NAB2 counteracts WT1-mediated repression of the *IRF8* promoter A reporter plasmid containing a 970 bp fragment of the *IRF8* promoter [31] was transfected into 293T/17 together with an expression plasmid encoding WT1 and different amounts of *NAB2* expression vector, as indicated. Fortyeight hours after transfection, cell lysates were collected and analyzed for luciferase activity. Shown are normalized firefly luciferase levels, relative to those obtained with promoter reporter only (control). Mean values, bars ±S.E.M., *n* = 3. Stars indicate statistical significance (^*^: p < 0.05; ^***^: p < 0.001). Western blot (WB) shows increasing amount of expressed NAB2. Histone H3 (H3) as equal loading control.

To investigate functional consequences of a NAB2/WT1 interaction on WT1-dependent transcription, we performed promoter-reporter experiments. WT1 binds to the *IRF8* promoter and represses transcription of *IRF8* in leukemic cells [31]. To investigate the effect of NAB2 on this repression, a luciferase reporter vector containing the *IRF8* promoter with a WT1 responsive element [31] was transfected into 293T/17 cells together with *WT1*+/− and increasing amounts of *NAB2*. As expected, *WT1* repressed transcription (Figure 7), while *NAB2* alone showed no significant effect. Interestingly, cotransfection with *NAB2* partially reversed the WT1-mediated repression in a dose-dependent fashion (Figure 7), indicating that NAB2 indeed interferes with the transcriptional function of WT1.

### WT1 recruits NAB2 to the *IRF8* promoter

**Figure 8:**
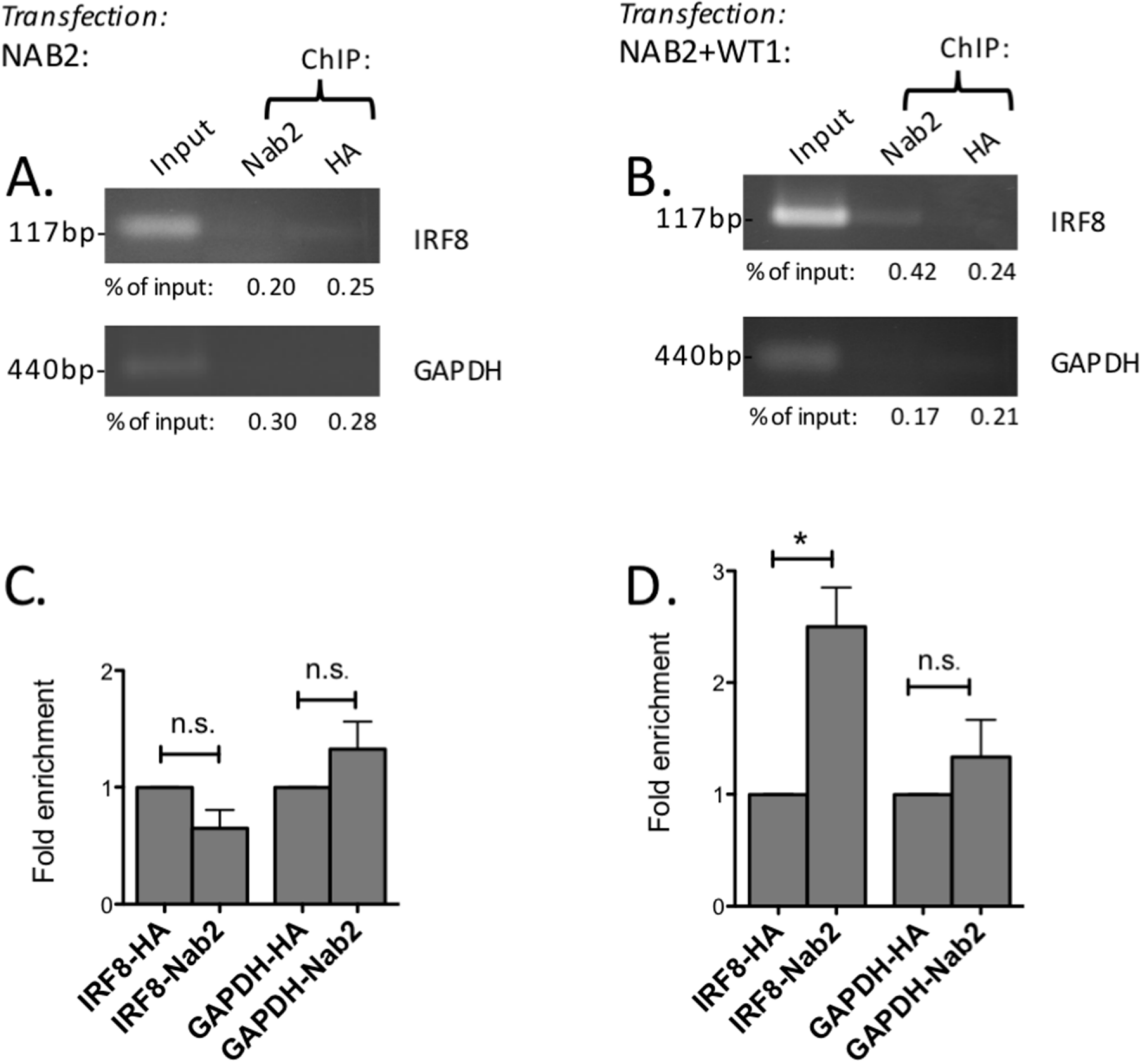
WT1 recruits NAB2 to the *IRF8* promoter 293T/17 cells were transfected with *NAB2* (A, C) or with *NAB2* plus *WT1* (B, D). Nuclear extracts were obtained after 48 hours and cross-linked chromatin was prepared. **(A, B)** Chromatin immunoprecipitation (ChIP) using an antibody against NAB2 (Abcam, ab135665) or with anti-HA antibody as negative control was performed. PCR amplification of precipitated DNA was done with primers specific for the *IRF8* promoter or with primers for the *GAPDH* promoter as negative control. Percentage of input, as determined by densitometry, shown as mean values, *n* = 3. **(C, D)** Fold enrichment as determined by densitometry. Mean values, bars ±S.E.M., *n* = 3. Star indicates statistical significance (^*^: p < 0.05), *n.s.*: not significant.

As WT1 binds directly to the proximal promoter of the *IRF8* gene [31], we now went on to investigate whether NAB2 binds the *IRF8* promoter in a WT1-dependent way. To this end, ChIP experiments were performed in 293T/17 cells, which have low expression of endogenous *WT1* and *NAB2*, thus allowing manipulation of WT1 and NAB2 levels by overexpression. Upon overexpression of *NAB2* only, no binding of NAB2 to the *IRF8* promoter was seen (Figure 8A, C). However, upon cotransfection of both *NAB2* and *WT1*, precipitation with anti-NAB2 antibody resulted in enrichment of the *IRF8*-promoter signal, as compared to signal obtained after precipitation with anti-HA, used as negative control (Figure 8B, D). These results indicate that WT1 can recruit NAB2 to the *IRF8*-promoter *in vivo*.

### Overexpression of *NAB2* increases expression of endogenous *IRF8*

**Figure 9:**
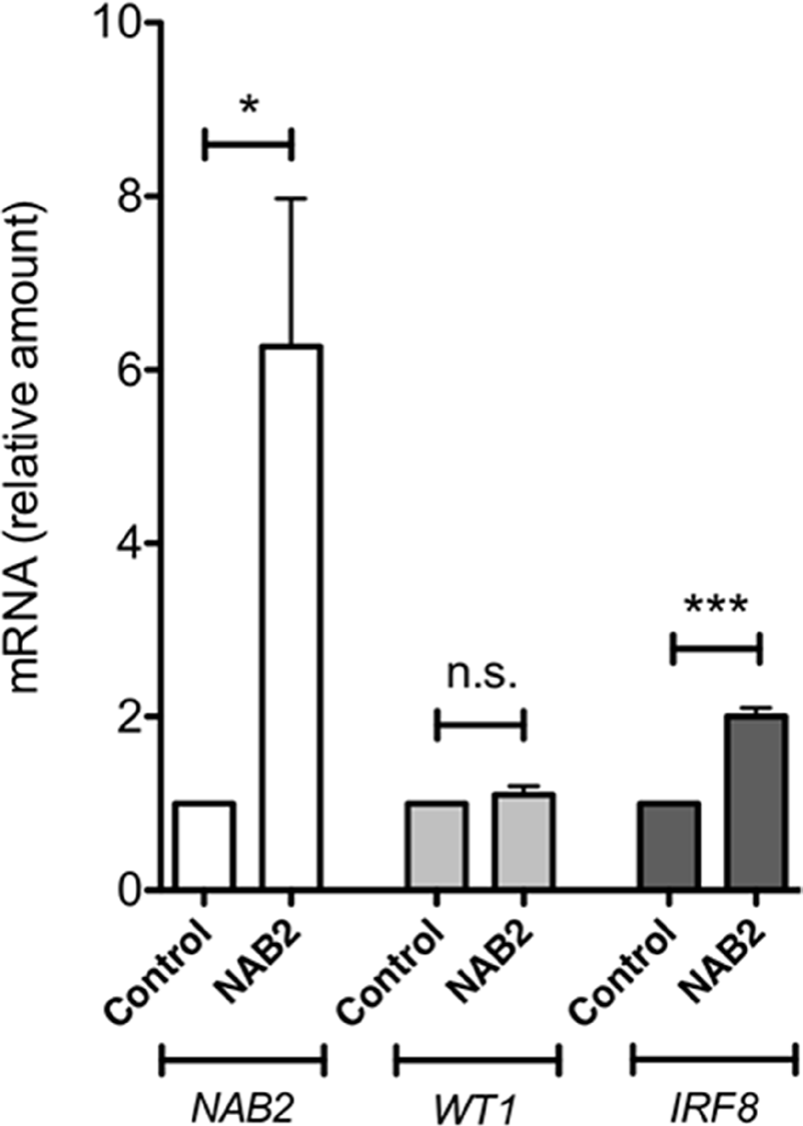
NAB2 overexpression in K562 cells enhances expression of endogenous IRF8 K562 cells were transfected with NAB2, or with empty vector, by electroporation, after which transformed cells were selected for by incubation of cells with puromycin or G418, as described in Material and Methods. After two weeks of selection, mRNA levels were analyzed by qPCR. Shown are levels of *NAB2* mRNA (open bars), *WT1* mRNA (light grey bars), and *IRF8* mRNA (dark grey bars), in pools of K562 cells overexpressing NAB2, normalized to levels in control cells transfected with empty vector. Mean values, bars ±S.E.M., *n* = 6. Stars indicate statistical significance (^***^: p < 0.001; *n.s*.; not significant).

**Figure 10:**
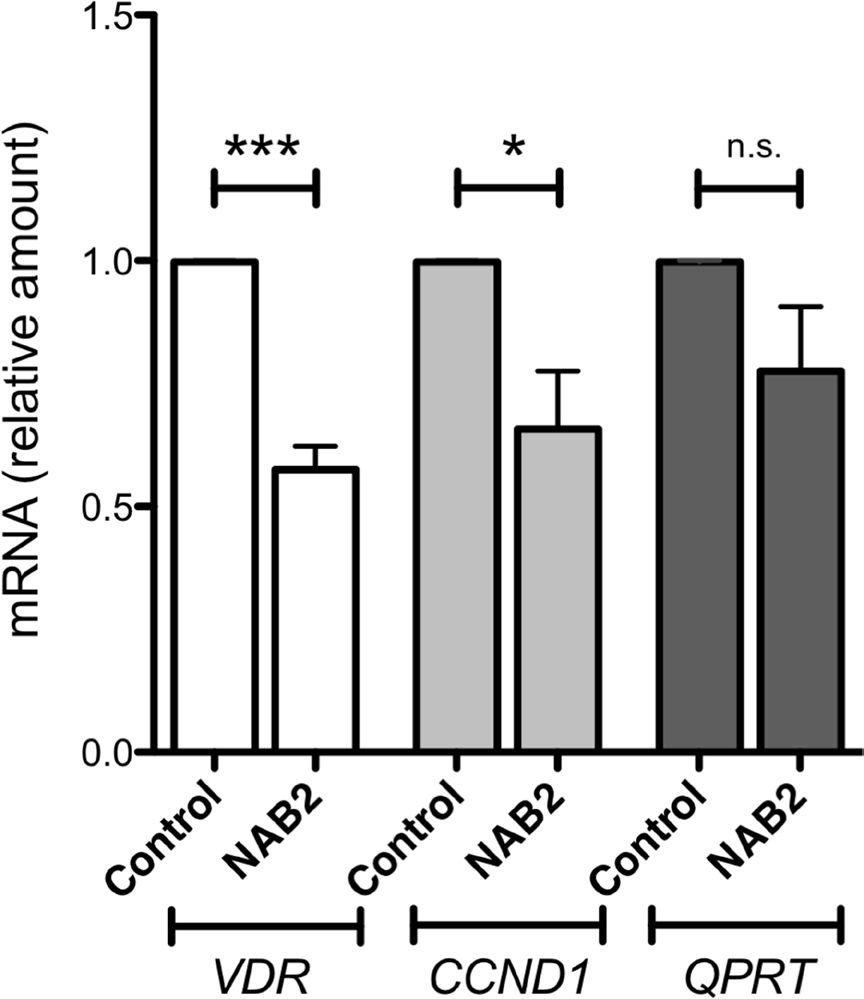
Effects of NAB2 overexpression in K562 cells on expression of the WT1-target genes *VDR, CCND1 and QPRT* K562 cells were transfected with NAB2, or with empty vector, by electroporation, after which transformed cells were selected for by incubation of cells with puromycin or G418, as described in Material and Methods. After two weeks of selection, mRNA levels were analyzed by qPCR. Shown are levels of *VDR* mRNA (open bars), *CCND1* mRNA (light grey bars), and *QPRT* mRNA (dark grey bars), in pools of K562 cells overexpressing NAB2, normalized to levels in control cells transfected with empty vector. Mean values, bars ±S.E.M., *n* = 4. Stars indicate statistical significance (^***^: p < 0.001; ^*^: p < 0.05; *n.s*.: not significant).

To investigate the effect of NAB2 on the expression of endogenous *IRF8*, we overexpressed *NAB2* in K562 cells, after which effects on levels of *WT1* and *IRF8* mRNA were analyzed. As expected, the levels of *NAB2* mRNA were raised in *NAB2*-transfected cells (Figure 9). While the amount of *WT1* mRNA was not affected, the expression of *IRF8* was clearly raised (Figure 9). These data are consistent with the notion that NAB2 can counteract a WT1-mediated repression of *IRF8* in leukemic cells. To extend this analysis into other WT1 target genes, the mRNA levels of the vitamin D receptor (*VDR*), cyclin D1 (*CCND1*) and quinolinate phosphoribosyltransferase (*QPRT*) were also analyzed. *VDR* and *QPRT* are directly transactivated by WT1 [32, 33] and by interacting with STAT3, WT1 enhances expression of *CCND1* [34]. As shown in Figure 10, expression of both *VDR* and *CCND1* were reduced in response to overexpression of NAB2, while the reduction of *QPRT* expression did not reach statistical significance. These results are consistent with the notion of NAB2 as a transcriptional comodulator of WT1 on several target genes.

## DISCUSSION

Although initially described as a suppressor gene in Wilms’ tumors, the transcription factor WT1 acts as an oncoprotein in hematopoietic malignancies [12]. In this work, we demonstrate that the transcriptional coregulator *NAB2* is transcriptionally upregulated by WT1 and that NAB2 protein binds to and modulates the transcriptional activity of WT1. These conclusions are based on our findings that (i) overexpression of *WT1* results in increased expression of *NAB2*, while suppression of *WT1* reduces *NAB2* levels, (ii) WT1 binds to the *NAB2* promoter, and (iii) that WT1 binds and recruits NAB2 to the WT1-target gene *IRF8*, reducing the repressive effect of WT1 on *IRF8* expression. Moreover, we discovered a positive correlation between the expression of *WT1* and *NAB2*, as well as a non-zero partial correlation, in leukemic gene expression data sets. Our conclusion, that *NAB2* is a target gene of WT1 in leukemic cells, is consistent with data from a global genome screening, indicating that WT1 binds to the *NAB2* gene in a Wilms' tumor cell line [35]. However, it should be noted that it cannot be ruled out that also other regulatory regions of the *NAB2* gene, outside the proximal promoter, may contain response elements to WT1.

The amino terminal part of WT1 contains domains that mediate transcriptional regulation, including separate suppression and activation domains [27]. Accordingly, WT1 can have a positive, as well as a negative, effect on transcriptional activity, dependent on the promoter and the cellular context. Some transcriptional coregulators bind directly to WT1, such as BASP1 [36] and FHL2 [37], acting as corepressor and coactivator of WT1, respectively. The coregulator PAWR (Par-4) binds to the zinc-fingers of WT1, thus inhibiting transcriptional activation [38], but also binds to the amino terminal of WT1, mediating transcriptional activation of WT1 [39]. This report adds NAB2 as a novel cofactor of WT1.

*NAB2* is transcriptionally induced also by the transcription factor EGR1 [24]. The NAB2 protein acts in a negative feed-back loop by inhibiting the transcriptional activity of EGR1 [24, 25]. At least two separate repression domains are present within NAB2, one of which interacts with the NuRD (nucleosome remodeling and deacetylase) chromatin-remodeling complex [40]. However, NAB2 can also stimulate EGR1-directed transcription [26]. The exact mechanism by which NAB2 modulates the transcriptional activity of WT1 remains to be investigated, but our data indicate that NAB2 can counteract the repressive effect of WT1 on the leukemic suppressor gene *IRF8*, suggesting that NAB2 potentially could modulate the oncogenic effects by WT1 in leukemia. The notion of NAB2 having a role in regulation of hematopoiesis is consistent with a previous report that NAB2 cooperates with EGR2 in suppression of the transcription factor GFI1 during neutrophil differentiation [41]. Our observation that overexpression of *NAB2* reduces expression of two other WT1 target genes, *VDR* and *CCND1*, further suggests that NAB2 can modify the effects of WT1 on several target genes.

In conclusion, we report *NAB2* as a novel target gene and a transcriptional coregulator of WT1 in hematopoietic cells. Whether NAB2 is a potential therapeutic target in leukemia remains to be determined.

## MATERIALS AND METHODS

### Cells and cell culture

K562, U937, and 293T/17 cells (DSZM, German Collection of Microorganisms and Cell Cultures, Braunschweig, Germany) were maintained in RPMI-1640 (K562, U937) or DMEM (293T/17) cell culture medium (Gibco Ltd., Paisley, United Kingdom) supplemented with 10% fetal calf serum (Gibco Ltd.). Cell lines were not passaged for more than 6 months. Umbilical cord blood after full-term deliveries was donated after informed consent, and mononuclear cells were isolated using Lymphoprep (Nycomed Pharma, Oslo, Norway), followed by enrichment of CD34^+^ cells with the CD34^+^ Progenitor Cell Isolation Kit (Miltenyi Biotec, Bergisch-Gladbach, Germany) according to the manufacturer's recommendations. CD34^+^ cells were maintained in StemSpan SFEM medium supplemented with 20% fetal calf serum and StemSpan™ CC100 (Stemcell Technologies, Vancouver, Canada).

### Vectors

Retroviral vectors for *WT1*+17AA/-KTS isoform and for the *WT1*+17AA/delZ construct (encoding a truncated WT1 protein without the zinc finger region) have been described previously [42, 43]. *NAB2*-OmicsLink expression vector (pEZ-M68-*NAB2*, #EX-S0519-M68) and *NAB2* promoter-reporter vector (HPRM19356-PG04) were purchased from GeneCopoeia (Rockville, MD, USA). Expression vector for *WT1*+17AA/-KTS isoform (pCMV-CB6-WT1+/−) and for *EGR1* (pCMV5-Egr-1) were kind gifts from Dr. Rauscher III, Philadelphia, USA. The shRNA lentiviral vector targeting *WT1* (with TRC2-pLKO-puro backbone) [28] was obtained through the Addgene non-profit plasmid repository (https://www.addgene.org). Scrambled shRNA lentiviral vector was from Sigma-Aldrich (St Louis, MO, USA).

### Viral transduction, electroporation and RNA extraction

Retroviral particles were produced as previously described [43]. Lentiviral particles were produced at the core facility Vector Unit, Lund University. For transduction of cells, non-tissue culture-coated well plates were coated with RetroNectin (Takara Clontech, Otsu, Shiga, Japan) for two hours at room temperature, blocked with 2% bovine serum albumin for 30 minutes at room temperature, and then coated with virus-containing medium for one hour at 4°C under centrifugation. Cells were cultured in the virus-coated wells for 48 hours, after which retrovirally transduced cells, expressing GFP from an IRES, were selected by sorting on a FACS Aria flow cytometer (BD Biosciences Immunocytometry Systems, San José, CA, USA). Lentivirally transduced cells were selected for by adding 1.5 μg/ml puromycin to the culture 48 hours after transduction, followed by incubation for 72 hours. U937 cells were transfected by electroporation using a Bio-Rad Gene Pulser II System (settings 300 V, 960 μF and a 0.4 cm cuvette) with *NAB2* promoter-reporter vector (HPRM19356-PG04), pCMV-CB6-WT1+/− and pCMV5-Egr-1, as indicated. K562 cells were transfected by electroporation, with settings as for U937 cells, with pEZ-M68-NAB2, or with pcDNA3 as control. After 48 hours, puromycin (1 μg/ml) or G418 (1.5 mg/ml), respectively, was added. Transfected cells were selected for in the continued presence of puromycin for two weeks. Total RNA was extracted from cells using the RNeasy Mini Kit (Qiagen GmbH, Hilden, Germany), according to the manufacturer's recommendations.

### Immunoblotting

Immunoblotting was performed as previously described [44]. Briefly, membranes were blocked using 5% dry milk in TBS-T buffer, followed by incubation overnight with either of the following primary antibodies: rabbit anti-WT1 polyclonal antibody (Santa Cruz Biotechnology, Dallas, TX, USA, clone C-19, sc-192) at dilution 1:1,000 in 0.5% dry milk in TBS-T buffer, mouse anti-NAB2 monoclonal antibody (Santa Cruz Biotechnology, clone 1C4, sc-23867) at dilution 1:1,000 in 5% dry milk in TBS-T buffer, or mouse anti-GAPDH monoclonal antibody (Santa Cruz Biotechnology, clone 6C5, sc-32233) at dilution 1:3,000 in 0.5% dry milk in TBS-T buffer. The membrane was then incubated with secondary antibody (goat anti-mouse-HRP conjugate or goat anti-rabbit-HRP conjugate at dilutions 1:3,000 in 0.5% dry milk in TBS-T buffer; all secondary antibodies purchased from Bio-Rad, Hercules CA, USA) for one hour at room temperature, after which expression was analyzed using the EZ-ECL kit (Biological Industries, Kibbutz Beit Haemek, Israel) and a ChemiDoc MP system (Bio-Rad). Calculations were made using BioRad's Image Lab software v 5.2.

### Real-time qPCR

Reverse transcription PCR was performed using the High Capacity cDNA kit (ThermoFisher Scientific, Waltham, MA, USA) according to manufacturer's instructions. Quantitative PCR (qPCR) was done using TaqMan Gene Expression Assays, on a StepOne Plus Real-Time PCR system (Life Technologies, Carlsbad, CA, USA). Primer-probe pairs used for *WT1* (Hs00240913_m1); *NAB2* (Hs00195573_m1); *IRF8* (Hs00175238_m1); *GAPDH* (Hs99999905_m1); *VDR* (Hs 01045843_m1); *CNND1* (Hs 00277039_m1); and *QPRT* (Hs 00204757_m1) were purchased from Applied Biosystems (Foster City, CA, USA) as assays-on-demand. Data were collected and analyzed using the Applied Biosystems StepOne^TM^Real-Time PCR Software v2.0. The relative quantification in gene expression was determined using the ΔΔCt method [45].

### Gene expression correlations

We used microarray data from NCBI GEO, accession numbers GSE22056 (adult and pediatric AML, *n* = 625), GSE21261 (adult AML, *n* = 408), GSE12417 (adult AML, *n* = 242), GSE13159 (adult AML and CML, *n* = 2,096), GSE6891 (adult AML, *n* = 461). GSE14468 (adult AML, *n* = 524), GSE10358 (adult AML, *n* = 304), GSE15434 (adult AML, *n* = 251), GSE17855 (pediatric AML, *n* = 237), and GSE22845 (adult AML, *n* = 154). All data were normalized to a log-normal distribution. To compute the correlation between *WT1* and *NAB2*, we used Pearson correlation coefficient test. Additionally, we tested for partial correlations between *WT1* and *NAB2* in 3,844 acute myeloid leukemia (AML) patients (GSE accession numbers GSE6891, GSE7757, GSE10358, GSE12417, GSE12662, GSE13159, GSE14468, GSE15061, GSE15434, GSE17855, GSE21261, and GSE22056) using the Ultranet tool [29] with the lambda 0.1 setting to find possible gene network partners of *WT1*. Ultranet computes partial correlation, minimizing indirect correlation exerted through other variables in the correlation matrix.

### Reporter experiments

For *NAB2* promoter analyses a promoter-reporter vector containing the 1,300 bp proximal *NAB2* promoter (HPRM19356-PG04) was transfected by electroporation into U937 cells, alone or together with expression vector for WT1+/− or for EGR1. Secreted alkaline phosphatase, expressed from the reporter plasmid, was used for transfection normalization. After 48 hours, luciferase assay was performed using the Secrete-PairTM Dual Luminescence Assay Kit (Genecopoeia). For *IRF8*-promoter analyses, a reporter plasmid containing the 970 bp proximal promoter of the *IRF8* [31] was transfected with WT1+/− and with increasing amount of *NAB2* expression vector into 293T/17 cells, using Attractene transfection reagent (Qiagen, Hilden, Germany). Cotransfection with a Renilla-luciferase reporter was used for transfection normalization. After 48 hours, luciferase assay was performed using the Dual luciferase reporter assay kit (Promega, Madison, WI, USA) and a TD20/20 luminometer (Turner Designs, Sunnyvale, CA, USA) according to the manufacturers’ instructions.

### ChIP-PCR

Chromatin Immunoprecipitation (ChIP) experiments were performed using the Chromatin Immunoprecipitation (ChIP) Assay Kit (Millipore, Darmstadt, Germany) according to the manufacturer's protocol. Briefly, cross-linked chromatin was prepared from K562 or 293T/17 cells transfected with *WT1* and/or *NAB2*. The mouse anti-WT1 monoclonal antibody (F-6) was from Santa Cruz Biotechnology, while the rabbit polyclonal antibody anti-NAB2 (ab135665) was from Abcam (Cambridge, U.K.). ChIP samples were analyzed by qPCR as previously described [46], using 2 μl of immunoprecipitated material and 1 μl of input control diluted 1/30 as templates. Specific primers for a WT1-binding region in the *NAB2* promoter were 5′-cacctcggtccccaattc-3′ (forward) and 5′-gcttagagactgggagagg-3′ (reverse) (Figure 3); for the WT1-binding region −52 to −38 in the *IRF8* promoter primers were 5′-ttctcggaaagcagagcacttc-3′ (forward) and 5′-gccttaaaaagggtcgtggg-3′ (reverse) [31]; and in the *GAPDH* promoter (used as negative control) 5′-ggtcgtattgggcgcctggtcacca-3′ (forward) and 5′-cacacccatgacgaacatgggggc-3′ (reverse) [46]. Quantification of qPCR was determined by densitometry using ImageJ software (http://rsbweb.nih.gov/ij/download.htlm). Fold enrichment was calculated as the signal over background, obtained with an unrelated antibody, as described [46].

### Coimmunoprecipitation

293T/17 cells were transfected with *WT1*, or with *WT1* in combination with *NAB2*, using Attractene Transfection Reagent (Qiagen) according to manufacturer's protocol. Fortyeight hours post transfection, nuclear extracts were prepared using the Nuclear Complex Co-IP kit (Active Motif, Carlsbad, CA, USA) according to manufacturer's protocol. Briefly, cells were washed with a PBS/Phosphatase inhibitors solution, harvested, and pelleted at 430 × *g* and 4°C for 5 minutes. The pellet was resuspended in 1 × hypotonic buffer. After 15 minutes of incubation on ice, the detergent was added and the suspension pelleted at 14,000 × *g* for 30 seconds at 4°C. The nuclei were then extracted by resuspension in digestion buffer and shearing enzymes were added. After 90 minutes of incubation at 4°C, EDTA was added. The suspension was then centrifuged for 10 minutes at 14,000 × *g* at 4°C and the supernatant was saved for protein quantification by Bradford protein assay. To 500 μg of nuclear extract, 5 μl of rabbit polyclonal anti-WT1 (Santa Cruz Biotechnology, clone C-19, sc-192) or mouse anti-NAB2 monoclonal antibody (Santa Cruz Biotechnology, clone 1C4, sc-23867) was added and mixed with 500 μl 1 × High IP buffer. After incubation overnight during rotation at 4°C, antibody-bound proteins were captured using a Protein G Agarose Column (Active Motif) and washed (1 × High IP Wash buffer) and subsequently eluted with 2 × Reducing buffer. Pure glycerol (Sigma-Aldrich) was added to each elution. The samples were boiled and thereafter subjected to SDS-PAGE followed by immunoblotting, using rabbit anti-WT1 polyclonal antibody (Santa Cruz Biotechnology, clone C-19, sc-192) or rabbit anti-NAB2 polyclonal antibody (Abcam, Cambridge, UK, ab135665). To eliminate detection-interference from heavy-chain and light-chain IgG-fragments, the Clean-blot IP detection kit (HRP) (Thermo Scientific, Rockford, IL, USA) was utilized, according to manufacturers' instructions.

Coimmunoprecipitation of endogenous WT1 and NAB2 proteins was performed with nuclear extracts from K562 cells, prepared as described above, but with the use of 1 × Low IP buffer for immunoprecipitation, and washing of the immunoprecipitate with 1 × Low Wash buffer with no DTT nor NaCl added, for lower stringency, according to the manufacturers' instructions. Elution was as described above and eluted material was separated by SDS-PAGE (20% of positive control loaded). Immunoblotting with anti-NAB2 was performed as described above.
